# Right-hemisphere (spatial?) acalculia and the influence of neglect

**DOI:** 10.3389/fnhum.2014.00644

**Published:** 2014-08-20

**Authors:** Silvia Benavides-Varela, Marco Pitteri, Konstantinos Priftis, Laura Passarini, Francesca Meneghello, Carlo Semenza

**Affiliations:** ^1^Neuropsychology Unit, IRCCS Fondazione Ospedale San CamilloVenice, Italy; ^2^Department of General Psychology, University of PadovaPadova, Italy; ^3^Neuroscience Department, University of PadovaPadova, Italy

**Keywords:** right hemisphere damage patients, calculation deficits, multiplication, subtraction, spatial acalculia

## Abstract

The present study aimed at exploring basic number and calculation abilities in right-hemisphere damaged patients (RHD), focusing primarily on one-digit orally presented tasks, which do not require explicit visuo-spatial abilities. Twenty-four non mentally-deteriorated RHD patients [12 with clinical neglect (RHDN+), 12 without clinical neglect (RHDN−)], and 12 healthy controls were included in the study. Participants were administered an *ad hoc* numerical battery assessing abilities such as counting, number magnitude comparison, writing and reading Arabic numerals and mental calculation, among others. Significant differences emerged among healthy controls and both the RHDN+ group and the RHDN− group, suggesting that the mathematical impairment of RHD patients does not necessarily correspond to the presence of left-neglect. A detailed analysis of the sub-tests of the battery evidenced expected differences among RHDN+ patients, RHDN− patients, and controls in writing and reading Arabic numerals. Crucially, differences between RHDN+ patients and controls were also found in tasks such as mental subtraction and mental multiplication, which do not require written visuo-spatial abilities. The present findings thus suggest that unilateral right hemisphere lesions may produce specific representational deficits that affect simple mental calculation, and not only the spatial arrangement of multi-digit written numbers as previously thought.

## Introduction

The first to point out a possible contribution of the right hemisphere to calculation was Henschen (1926). He argued that the right hemisphere contributed to calculation only in a compensatory manner, when a lesion in the left hemisphere is very large. A couple of decades later, Goldstein (1948) still believed that there was no proof that the minor hemisphere had any role in calculation. Likewise, Sperry et al. (1969) argued from the study of split-brain patients that the capacity for calculation of the right hemisphere is “almost negligible.”

To this day, acalculia following a right hemisphere lesion is a very poorly defined entity. It is consistently found in group-studies, though in various proportions partly due to the different sensitivity of the calculation tests used to assess patients. Moreover, most studies appear incomplete because they excluded neglect patients, and some considered only retrorolandic patients.

According to Hécaen and Angelergues (1961), who only considered retrorolandic lesions, the alexia/agraphia variety occurred in 8% of the cases, anarithmetria occurred in up to 15% of the cases, whereas calculation disorders of spatial type were present in 75% of patients with right retrorolandic lesions. Further figures for the incidence of acalculia in right-hemisphere lesions appear in Hécaen (1962): alexia/agraphia for numerals is found in 2.1% of the lesions; anarithmetria in 20.2% and spatial acalculia in 31% of cases.

Grafman et al. comparing left and right-hemisphere-damaged (RHD) patients with anterior and posterior lesions, concluded that patients with left posterior lesions performed exceedingly worse than other patients, but the right posterior group was much worse than both the two anterior groups (Grafman et al., 1982). These results persisted after correction of acalculia scores by the results of other neuropsychological tests (e.g., Raven's progressive matrices, Token Test, Crosses Test, and assessment of constructional apraxia). Grafman et al. concluded that, even if different factors (like impairment of intelligence, visuo-constructive difficulties, and aphasia) may contribute to calculation disorders, acalculia can still be partially independent from such disorders. They were surprised to find that both patients with right-hemisphere lesions and patients with Wernicke's aphasia showed spatial disorders; spatial disorders in Wernicke's aphasia could not be secondary to language impairment. The nature of errors in RHD patients was not discussed.

Ardila and Rosselli (1994) tested 21 RHD patients: 6 prerolandic and 15 retrorolandic. Their findings suggested that acalculia appeared particularly evident in written calculation and would be better preserved in mental calculation. In reading and writing numbers, “spatial” alexia and agraphia led to particular errors: feature and digit addition, inability to use the spaces to join and separate numbers, difficulty in maintaining the written line in a horizontal position, increased left margins and unsteadiness in maintaining left margins, disrespect of spaces and spatial disorganization of written material. As far as the calculation system was concerned, Ardila and Rosselli (1994) found loss of calculation automatisms and reasoning errors; however, these errors may not be specific to right-hemisphere damage. Impossible results were not rejected.

Using an extensive battery, Basso et al. studied number and calculation deficits in 26 RHD patients, critically excluding patients affected by left neglect (LN) (Basso et al., 2000). In this study only 3 out of 26 patients were classified as acalculic. The exact data from these three patients on written calculation were not reported.

On the other hand, single case studies of acalculia in RHD patients are rare and mostly anecdotal (e.g., Leleux et al., 1979; Ardila and Rosselli, 2002). The only detailed single case study is probably that reported by Granà et al. (2006). An important portion of their patient's errors could be better explained as spatial in nature and specifically related to the demands of a multi-digit multiplication. Granà et al. suggested that these errors reflected difficulties in relying on a visuo-spatial store containing a layout representation specific to multiplication. Thus the patient, while knowing what, when, and how to carry out the various steps, did not know where (Granà et al., 2006). There are no other single case reports of this specific problem. This deficit needs to be carefully looked for and convincingly distinguished from other types of deficits through a very patient analysis. It is not surprising, therefore, that it is not easily revealed with a standard assessment.

Neuroimaging studies evidenced a number of mathematical abilities sustained by the right hemisphere (Dehaene et al., 2003, 2004; Salillas and Semenza, in press). Bilateral activation, at least of the parietal lobes, is the most common finding in neuroimaging studies on calculation. Evidence of relatively more intense activation on the right hemisphere seems to be possibly related to proficiency (Zago et al., 2001). A meta-analysis conducted on functional MRI-based studies (Arsalidou and Taylor, 2011) found that neural activity is dominant in the left hemisphere for addition, either bilaterally or in the right hemisphere for subtraction, and primarily in the right-dominant hemisphere for multiplication. Consistently with this finding, Rosenberg-Lee et al. (2011) found that multiplication evokes significantly greater activation in the right posterior intraparietal sulcus (Rosenberg-Lee et al., 2011). More recently, Price et al. (2013) also demonstrated the role of the right hemisphere in math learning, insofar as a greater activation in the right intraparietal sulcus during calculation was related to lower math scores (reflecting different strategies by less competent calculators) (Price et al., 2013).

Using transcranial magnetic stimulation (TMS), several studies have shown that the right hemisphere was related to specific numerical abilities (for a review, see Salillas and Semenza, in press). For instance, Andres et al. (2011) found that disruption to both the left and right horizontal intraparietal sulci leads to impaired multiplication. Interestingly, frequent errors of the retrieval type (table results other than the target) suggested that bilateral disruption of the horizontal intraparietal sulcus impaired retrieval processes (Andres et al., 2011). Salillas et al. (2012) further showed that efficiency in simple multiplication depends on the ventral region of the intraparietal sulcus in the right hemisphere (Salillas et al., 2012), considered to be critical for motion representation and automatization (Salillas et al., 2009).

Recent studies conducted with direct cortical electrostimulation (DCE) also suggested some role of the right hemisphere in simple calculation. Yu et al. (2011) found that stimulation of the right parietal lobe impaired a patient's performance on simple subtraction problems. They related the right parietal involvement in subtraction to the involvement of quantity processing, rather than verbal numerical processing. They also assumed a role of spatial representations of numbers in the selected parietal sites. Della Puppa et al. (2013) provided, instead, the first DCE study that shows non-dominant right hemisphere involvement in multiplication (positive sites were found in the angular gyrus, the supramarginal gyrus, the interparietal sulcus, and the superior parietal lobule) and addition (in the supramarginal gyrus).

One conclusion from this perusal of the literature is that, while a role of the right hemisphere in calculation seems undeniable, the reason(s) why right hemisphere acalculic patients err the most, and, in particular, whether their deficits are secondary to visuo-spatial functions is still very unclear. A further group study is needed, on all types of focal right lesions (with neuroimaging evidence), on a wide range of math tasks, relating the results to several different abilities and neuropsychological deficits. The purpose of the present study is more limited. The issue is addressed here, for the first time, of how and to what extent LN contributes to right-hemisphere acalculia.

## Materials and methods

### Participants

#### Control group

Twelve healthy participants (5 males and 7 females) matched for age (mean 59.6 years; SD 7.8; range: 46–72) and education level (mean 9.7 years; SD 4.3; range: 5–17) with RHD patients were enrolled in the present study. All healthy participants had no history of neurological or psychiatric illness.

#### Patients

Twenty-four patients (15 males and 9 females) who had suffered right-hemisphere stroke were included in the study. Inclusion criteria were absence of dementia, substance abuse, and psychiatric disorders. All patients were right-handed and had unilateral right-hemisphere stroke lesions documented by CAT or MRI scans (actual images, that would have allowed lesion mapping, were, unfortunately, not available for all participants). In no case a left hemisphere lesion was reported. Patients were tested with the Behavioral Inattention Test, conventional part (BIT-C; Wilson et al., 1987), and they were subsequently divided in two groups, according to the BIT-C cut-off score (<130/146). There were 12 RHD patients with LN (RHDN+; scores < 130) and 12 RHD patients without LN (RHDN−; scores > 129). Demographic and clinical data are reported in Table 1.

**Table 1:** **Demographic and clinical data of all the participants**.

**Participant**	**Gender**	**Age (years)**	**Education (years)**	**Onset of illness (months)**	**Lesion type**	**Lesion location**
RHDN+_1	M	64	4	17	IS	Deep fronto-temporal
RHDN+_2	F	72	5	2	IS	Fronto-temporo-parietal
RHDN+_3	M	72	8	2	HS	Deep temporo-parietal
RHDN+_4	F	75	8	3	IS	Deep parieto-occipital
RHDN+_5	F	64	5	4	IS	Deep fronto-parietal
RHDN+_6	M	68	18	4	IS	Fronto-temporo-parietal
RHDN+_7	M	42	8	1	IS	Fronto-parietal
RHDN+_8	M	58	13	5	IS	Fronto-parieto-temporo-occipital extending to deep structures
RHDN+_9	F	45	8	6	IS	Fronto-temporo-parietal
RHDN+_10	F	66	13	15	IS	Fronto-temporo-parietal
RHDN+_11	F	72	5	67	IS	Temporo-parietal
RHDN+_12	M	70	5	7	HS	Deep fronto-parietal
Mean (*SD*)		64.0 (10.6)	8.3 (4.3)	11.1 (18.3)		
RHDN−_1	M	56	8	2	IS	Fronto-insulo-parietal
RHDN−_2	F	55	8	12	IS	Fronto-temporo-parietal
RHDN−_3	F	63	5	1	IS	Parietal
RHDN−_4	M	71	5	33	IS	Fronto-parietal
RHDN−_5	M	72	18	38	IS	Deep fronto-parietal
RHDN−_6	M	61	13	19	IS	Parietal
RHDN−_7	M	54	10	2	IS	Thalamic
RHDN−_8	M	66	18	48	IS	Deep fronto-parietal
RHDN−_9	F	57	5	51	IS	Deep fronto-parietal and caudate
RHDN−_10	M	54	8	1	IS	Deep fronto-temporal
RHDN−_11	M	69	5	11	IS	Deep para-thalamic
RHDN−_12	M	70	8	23	HS	Deep frontal and basal ganglia
Mean (*SD*)		62.3 (7.1)	9.2 (4.73)	20.1 (18.5)		
NHP_1	F	65	5	–	–	–
NHP_2	M	46	17	–	–	–
NHP_3	F	57	8	–	–	–
NHP_4	M	62	11	–	–	–
NHP_5	F	47	17	–	–	–
NHP_6	M	72	13	–	–	–
NHP_7	F	66	5	–	–	–
NHP_8	F	60	12	–	–	–
NHP_9	M	58	7	–	–	–
NHP_10	F	57	5	–	–	–
NHP_11	F	68	8	–	–	–
NHP_12	M	57	8			
Mean (*SD*)		59.6 (7.8)	9.7 (4.3)			

*IS, ischemic stroke; HS, haemorrhagic stroke; RHDN+, Right-hemisphere damaged patient with LN; RHDN−, Right-hemisphere damaged patient without LN; NHP, Neurologically healthy participant*.

There were no significant differences in age [*F*_(2, 33)_ = 0.8; *p* = 0.458] or education [*F*_(2, 33)_ = 0.28; *p* = 0.756] among the groups of patients and the group of healthy control participants. Moreover, the time since lesion did not differ significantly between the RHDN+ and RHDN− groups [*t*_(22)_ = 1.19; *p* = 0.244]. As a standard procedure, all patients also underwent an evaluation that included the Mini Mental State Examination (MMSE) test (Magni et al., 1996) to exclude general cognitive impairment (see Table 2).

**Table 2:** **Patients' performance on neuropsychological tests**.

**Patient**	**MMSE**	**BIT-C**	**Line crossing**	**Letter cancelation**	**Star cancelation**	**Figure and shape copying**	**Line bisection**	**Drawing**
RHDN+_1	23.4	95	36/36	11/40	40/54	1/4	6/9	1/3
RHDN+_2	23.3	55	16/36	23/40	15/54	0/4	0/9	1/3
RHDN+_3	23.4	115	36/36	24/40	52/54	1/4	1/9	1/3
RHDN+_4	26	129	36/36	39/40	47/54	1/4	6/9	0/3
RHDN+_5	24.9	92	36/36	32/40	20/54	1/4	3/9	0/3
RHDN+_6	25.2	55	18/36	16/40	21/54	0/4	0/9	0/3
RHDN+_7	24.9	107	36/36	26/40	37/54	1/4	6/9	1/3
RHDN+_8	23.2	97	26/36	37/40	33/54	1/4	0/9	0/3
RHDN+_9	27	112	31/36	38/40	36/54	1/4	5/9	0/3
RHDN+_10	22	69	24/36	23/40	20/54	1/4	1/9	0/3
RHDN+_11	24.3	84	18/36	31/40	28/54	1/4	6/9	0/3
RHDN+_12	27.3	111	29/36	29/40	47/54	0/4	6/9	0/3
RHDN−_1	25	135	34/36	34/40	54/54	3/4	7/9	3/3
RHDN−_2	30	143	36/36	40/40	54/54	3/4	9/9	1/3
RHDN−_3	27.9	143	36/36	40/40	54/54	4/4	7/9	2/3
RHDN−_4	25.3	138	36/36	36/40	54/54	3/4	9/9	0/3
RHDN−_5	20	139	36/36	37/40	53/54	4/4	7/9	2/3
RHDN−_6	26.2	145	36/36	40/40	54/54	4/4	8/9	3/3
RHDN−_7	24	141	36/36	38/40	54/54	3/4	9/9	1/3
RHDN−_8	26.2	139	36/36	38/40	52/54	2/4	8/9	3/3
RHDN−_9	30	139	36/36	36/40	53/54	3/4	9/9	2/3
RHDN−_10	26.2	142	36/36	38/40	54/54	3/4	9/9	2/3
RHDN−_11	26.9	142	36/36	40/40	54/54	2/4	8/9	2/3
RHDN−_12	19.4	144	36/36	39/40	54/54	4/4	9/9	2/3

The MMSE scores did not differ significantly between the RHDN+ and RHDN− groups [*t*_(22)_ = 0.958; *p* = 0.348]. The data analyzed in the current study were collected in accordance with the Helsinki Declaration II and the Institutional Ethics Committee of the IRCCS San Camillo Hospital Foundation, Lido-Venice, Italy. Prior to participation, all patients signed an informed consent form.

### Numerical screening

Participants were administered an *ad hoc* numerical battery (see Supplementary Material) for assessing counting abilities, odd/even number judgment, number magnitude comparison, writing Arabic numbers to dictation, reading Arabic numbers, recognition of arithmetical operations, one-digit mental multiplication, one-digit mental addition, one-digit mental subtraction, and number repetition. All tasks but reading and writing Arabic numbers were presented and solved orally. The maximum overall score was 137.

### Statistical analysis

The comparisons of the general performance on the Numerical Screening were carried out using the non-parametric Kruskal–Wallis test. This test was chosen because of the lack of homogeneity of variance among groups in the Numerical Screening as measured with Bartlett multiple-sample test (χ^2^_(2)_ = 44.03; *p* < 0.0001). *Post-hoc* comparisons between group-pairs were carried out using Bonferroni correction for multiple comparisons, set at a conservative error level of alpha = 0.016 (two-tailed).

Comparisons of the performance on the subtests of the Numerical Screening were carried out using two sample *t-tests* (two-tailed). The significance level was set according to Bonferroni's correction for multiple comparisons (alpha = 0.005).

In addition, we performed Pearson's correlation tests to determine the association between the patients' performance in number-related tasks and their visuo-spatial abilities as measured by the BIT-C. To make sure that the relations between those tests were not mediated by a general cognitive or demographic factor, we computed partial correlations, in which the impact of age, gender, education, and general cognitive efficiency (as measured by MMSE) were controlled for. Moreover, in order to evaluate the association between the numerical tasks and spatial attention, the Pearson's correlation analysis was performed with two subtests of the BIT-C (letter cancelation and start cancelation). Both cancelation tests assess the ability to visually scan an array and select appropriate responses while suppressing inappropriate ones; they have been used to measure attention (e.g., Casco et al., 1998) and are good predictors of spatial attention abilities in neuropsychological patients (Wilson et al., 1987).

## Results

### Performance of the groups on the numerical screening

**Figure 1:**
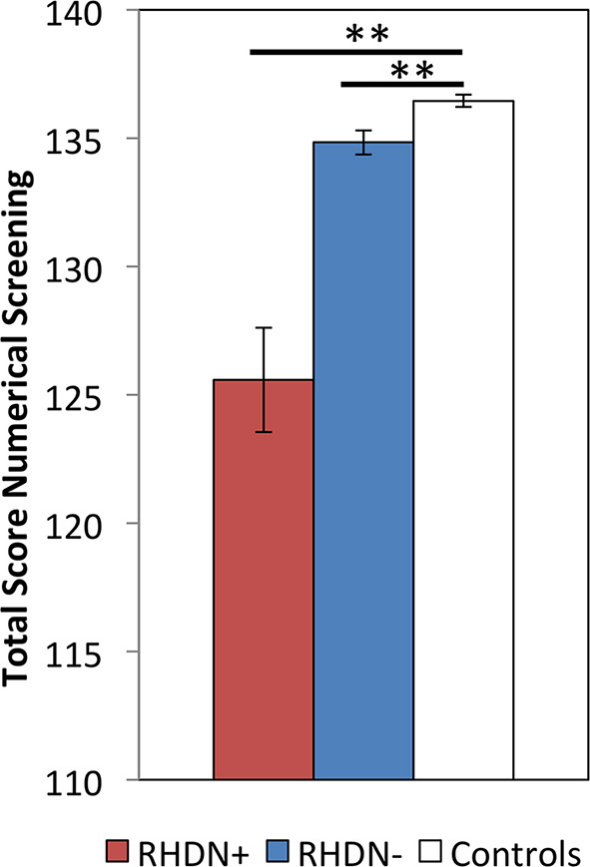
**Performance of the three groups: RHDN+, RHDN−, and healthy controls in the Numerical Screening Test**. Error bars indicate standard errors of the mean. ^**^p < 0.005.

The Kruskal–Wallis test revealed a significant effect of Group on the Numerical Screening [χ^2^_(2, *N* = 36)_ = 23.74; *p* < 0.0001]. *Post-hoc* comparisons, corrected with Bonferroni, showed significant differences between the RHDN+ group and neurologically healthy controls (*p* < 0.0001). There was also a significant difference between the RHDN- group and healthy controls (*p* < 0.005), and between RHDN+ and RHDN− groups (*p* < 0.0005). Figure 1 shows the results of the performance of groups on the task.

Similar results were obtained after excluding reading and writing numbers to dictation from the Numerical Screening, which were the only two tasks requiring explicit visuo-spatial abilities in the battery. After excluding these tasks there was a significant effect of Group [χ^2^_(2, *N* = 36)_ = 16.95; *p* < 0.0005], due to differences between the RHDN+ group and healthy controls (*p* < 0.0005), between the RHDN− group and healthy controls (*p* < 0.01), and between RHDN+ and RHDN− groups (*p* < 0.005).

### Comparison between patients and normal controls on the sub-tests of the numerical screening

Table 3 reports the results of the comparisons between patients and controls in the different sub-tests of the Numerical Screening. RHDN+ and healthy controls significantly differed in 3 sub-tests. Specifically, there were significant differences between RHDN+ and healthy controls in Writing Arabic numerals to dictation, mental multiplication, and mental subtraction (all *p*s < 0.05). RHDN+ and RHDN− differed significantly in Writing Arabic numerals to dictation. There were, however, no significant differences between RHDN− and healthy controls (all *p*s > 0.05). A detailed qualitative analysis of errors showed that RHDN+ patients' failure in writing Arabic numerals to dictation concentrated mainly on syntactic errors (96%); from these 91.30% were additions—of zeros—(73.91% to the rightmost edge of the number, 17.39% to the middle of the number), and 8.70% were omissions of digits (4.35% of the leftmost edge and 4.35% of an internal digit). The majority of multiplication errors were inside the same multiplication table (65.63%); 43.75% of incorrect responses were below the correct result whereas 56.25% were above. Patients' subtraction errors were below the correct answer in 70% of the cases. RHDN− patients showed considerably fewer errors in writing Arabic numerals to dictation (one eighth) with respect to RHDN+. From these, 66.6% were syntactic errors (33.3% additions and 33.3% omissions of an internal digit). RHDN− patients erred 43.75% less with respect to RHDN+ in mental multiplications. Like RHDN+ patients, the majority of RHDN−'s errors were inside the same multiplication table (77.78%). 61.1% of incorrect responses were above the correct result. Only one RHDN− patient failed a subtraction operation. Her answer evidenced a failure to apply the zero principle (*x* − *x* = 0).

**Table 3:** **Mean and statistical tests comparing patients and healthy controls in the different sub-tests of the Numerical Screening**.

**Task**	**NHP mean (*SD*)**	**RHDN+ mean (*SD*)**	**RHDN_Mean (*SD*)**	***t*-test RHDN+ vs. controls**	***t*-test RHDN_ vs. controls**	***t*-test RHDN+ vs. RHDN+**
Counting	4 (0)	3.5 (0.67)	4 (0)	*t*_(22)_ = 2.57; *p* = 0.017	*NA*; *n.s*.	*t*_(22)_ = 2.57;*p* = 0.017
Odd/even judgment	22 (0)	19.92 (2.97)	21.83 (0.39)	*t*_(22)_ = 2.43;*p* = 0.023	*t*_(22)_ = 1.48;*p* = 0.152	*t*_(22)_ = 2.22;*p* = 0.037
Number magnitude comparison	14 (0)	13.57 (0.45)	14 (0)	*t*_(22)_ = 1.91;*p* = 0.068	*NA*; *n.s*.	*t*_(22)_ = 1.92;*p* = 0.068
Writing arabic numerals to dictation	25 (0)	23 (1.71)	24.75 (0.45)	***t*_(22)_ = 4.06;*p* = 5 × 10^−4^**	*t*_(22)_ = 1.91;*p* = 0.068	***t*_(22)_ = 3.43;*p* = 0.002**
Reading arabic numbers	22 (0)	20 (2.95)	22 (0)	*t*_(22)_ = 2.34;*p* = 0.028	*NA*; *n.s*.	*t*_(22)_ = 2.35;*p* = 0.028
Recognition of operations	4 (0)	3.92 (0.29)	4 (0)	*t*_(22)_ = 1;*p* = 0.328	*NA*; *n.s*.	*t*_(22)_ = 1.00;*p* = 0.328
Mental one-digit multiplication	9.58 (0.67)	7.25 (1.66)	8.50 (1.17)	***t*_(22)_ = 4.52;*p* = 1 × 10^−4^**	*t*_(22)_ = 2.79;*p* = 0.011	*t*_(22)_ = 2.13;*p* = 0.044
Mental one-digit addition	9.92 (0.29)	9.25 (0.97)	9.83 (0.39)	*t*_(22)_ = 2.92;*p* = 0.03	*t*_(22)_ = 0.596;*p* = 0.557	*t*_(22)_ = 1.94;*p* = 0.065
Mental one-digit subtraction	10 (0)	9.17 (0.83)	9.92 (0.29)	***t*_(22)_ = 3.55;*p* = 0.002**	*t*_(22)_ = 1.00;*p* = 0.328	*t*_(22)_ = 2.94;*p* = 0.008
Number repetition	16 (0)	15.83 (0.39)	16 (0)	*t*_(22)_ = 1.48;*p* = 0.152	*NA*; *n.s*.	*t*_(22)_ = 1.48;*p* = 0.152

*Significance was set according to Bonferroni correction for multiple comparisons (Bold alpha < 0.005)*.

### Correlation between numerical screening tasks and visuo-spatial abilities in RHDN+ and RHDN− patients

The results of the Pearson's correlation analysis showed that visuo-spatial abilities, as measured by the BIT-C and by the performance on the Numerical Screening test, were intercorrelated in the group of 24 patients [*r*_(22)_ = 0.70;*p* < 0.001]. A further screening of the numerical sub-tests showed that only “Reading Arabic Numerals” correlated significantly with the BIT-C general score [*r*_(22)_ = 0.61;*p* < 0.005]. This correlation lost statistical significance after controlling for age, gender, education, and MMSE scores (ρ = 0.54;*p* = 0.01).

The patients' general score on the Numerical Screening test correlated significantly with both measures of spatial attention: the letter cancelation [*r*_(22)_ = 0.74;*p* < 0.001] and the starts cancelation [*r*_(22)_ = 0.61;*p* = 0.001]. Letter cancelation also correlated significantly with odd/even judgment [*r*_(22)_ = 0.63; *p* < 0.001]; recognition of arithmetical operations [*r*_(22)_ = 0.56; *p* = 0.004], and oral repetition [*r*_(22)_ = 0.59; *p* = 0.002]. All these correlations but recognition of arithmetical operations (ρ = 0.56; *p* = 0.01), and oral repetition (ρ = 0.55; *p* = 0.01) remained significant after age, gender, education, and MMSE were partial out.

All other correlations among the sub-tests of the Numerical Screening and the sub-tests of the BIT-C were not significant.

## Discussion

The present study explores basic numerical skills in RHD patients with and without LN. The results showed that patients performed significantly below the level of healthy controls on basic numerical tasks. The present findings suggest that unilateral right-hemisphere lesions can impair even simple number processing and calculation abilities. The observed impairment does not necessarily correspond to the presence of LN, nor to the sensorimotor and visuo-spatial disabilities of RHD patients. This reasoning finds support on at least four points: (a) the numerical deficit was evident in RHD patients, independently of the presence of LN—although the presence of LN seems to imply a more severe deficit—; (b) most tasks of the numerical battery did not explicitly require the use of sensorimotor abilities operating in the visuo-spatial domain; (c) when excluding reading and writing Arabic numerals from the battery, differences between both groups of patients and healthy controls persisted; (d) we found no correlation among the numerical screening tasks and patients' visuo-spatial abilities.

These results clearly indicate some level of dissociation between numerical deficits and the presence of LN. Still, the clinical diagnosis of LN appears to be associated with a clearer and more profound manifestation of the numerical deficit. In fact, the RHDN+ group showed an overall lower performance in the numerical battery with respect to the RHDN− group (Figure 1), with a more pronounced difference in the writing Arabic numerals to dictation task (Table 3). Indeed such task seems more prone to LN disturbances than the other tasks of the battery.

We also observed that RHDN+ patients showed specific impairments with respect to healthy controls in at least three tasks: writing Arabic numerals to dictation, mental multiplication, and mental subtraction. The pattern of errors in RHDN− patients was, instead, not specific to any of the tasks administered. Adding the errors obtained in the different tasks led to the difference found between the RHDN− patients and healthy controls in the total score of the battery, yet the tasks in which these errors appeared were not consistent across participants. The lack of significant differences in single tasks might be partly attributable to the ceiling performance of control and—though less so—RHDN− participants, which did not allow statistical comparisons in some of the tasks. For control participants there was also low variability in the total score of the battery due to ceiling effects. Evidently this screening battery (originally designed for clinical use and thus containing a limited number of items) is not very challenging for healthy participants. This restricts the power of statistical analysis and may have magnified the differences among controls and the other groups. The critical finding in this study however is that, notwithstanding the ease of the battery, RHD patients with or without neglect fail to reach the maximum score; and that RHDN+ show a specific profile of errors in this simplified battery.

Our findings converge with traditional literature on acalculia (Hécaen and Angelergues, 1961; Hécaen, 1962), previous neuropsychological studies (Ardila and Rosselli, 1994; Basso et al., 2000; Granà et al., 2006), neuroimaging findings (Dehaene et al., 2003, 2004; Rosenberg-Lee et al., 2011; Price et al., 2013), and cortical and transcranial stimulation reports (e.g., Göbel et al., 2001; Cohen Kadosh et al., 2007; Della Puppa et al., 2013; Salillas and Semenza, in press) arguing that the right hemisphere contributes significantly to number processing and calculation abilities.

The question arises as to the level over which the battery used for diagnosing LN in the present study drives our findings. In fact, the BIT-C does not specifically assess imaginal LN and it only assesses peripersonal LN, without looking at personal and extrapersonal spatial domains. Critical to the present study is the fact that LN extends to the scanning of mental images generated by the patients (Bisiach and Luzzatti, 1978), which may help explaining why in our study RHDN+ patients failed mental operations. Previous neuropsychological studies indicate that imaginal LN may indeed affect numerical representations. Zorzi et al. (2002) showed that when asked to indicate the midpoint of a drawn line, patients affected by LN placed the midpoint to the right of the actual middle. Interestingly, LN patients also misplaced the midpoint of a numerical interval when verbally asked to bisect it (e.g., stating that six is the numerical midpoint between one and nine). On the basis of these results, the authors suggested that LN in RHD patients may disrupt the mental representation of numbers (Zorzi et al., 2002). Similarly, a study by Vuilleumier et al. (2004) showed that when LN patients were asked to compare numbers with a reference one, they were slower at judging smaller numerals relative to a reference numeral than larger ones. For instance, when asked to judge whether numbers were smaller or larger than “5,” patients with LN were much slower to make a response to “4” compared with “6” and other higher numbers, but when asked to judge numbers as smaller or larger than “7” they were now much slower to respond to “6” compared with higher numbers. The authors interpreted these results suggesting that LN may produce specific representational deficits in number processing that implicate a difficulty to orient attention toward an internal representation (Vuilleumier et al., 2004). Thus, further exploring the correlations between batteries assessing specific subtypes of LN (particularly imaginal LN) and the patients' numerical (in particular calculation) deficits may be an important avenue for future research.

Still, our findings are compatible with the interpretation provided in the above-mentioned studies suggesting that patients with LN may experience an alteration in the mental representation of numbers. In addition, our data also suggest that such representational alteration may be associated with mathematical disabilities, in particular basic calculation deficits such as one-digit subtraction and multiplication. Interestingly, in the present study the qualitative analysis showed different patterns in multiplication and in subtraction errors. Whereas a greater amount of multiplication errors were overestimations of the correct result, subtraction errors were mainly underestimations. This may evidence differences in the strategies used to solve the two types of operations. Searching for the results of a multiplication may require forward scanning of products or multiplication tables, whilst subtraction entails backwards strategies or procedures. This may explain why the pattern of subtraction errors appears at first sight incompatible with the effects observed in previous studies (Zorzi et al., 2002; Vuilleumier et al., 2004), whereas both patterns of errors are rather consistent with a distorted representation affecting mainly the “left side” of the internal arrangement of numbers. Therefore, as anticipated in previous works (De Hevia et al., 2008; Salillas and Semenza, in press) besides numerical processing, mental representations impact some aspects of calculation, and/or the internally generated strategies required for it.

Although multiplication, and in particular retrieval of multiplication facts, has been traditionally associated with the left hemisphere (e.g., Dehaene and Cohen, 1997; Dehaene et al., 2003), our findings, showing a crucial involvement of the right hemisphere in mental multiplication and subtraction, should not be surprising. In a previous study, Andres et al. (2011) used fMRI-guided repetitive transcranial magnetic stimulation (rTMS) to address lateralization effects in the horizontal intra-parietal sulcus during simple arithmetic tasks. They found that stimulation in both the left and right hemispheres influenced simple subtraction and multiplication. Moreover, a subsequent study by Salillas et al. (2012) using single pulse TMS also evidenced that the right ventral region of the intraparietal sulcus was involved during multiplication. Thus, both studies, like ours, questioned the proposal of a left-lateralized network for arithmetic processing.

Our results are also compatible with a recent study evaluating the clinical impact of intra-operative cortical electro-stimulation on patients affected by right parietal brain tumor (Della Puppa et al., 2013). The study evidenced the involvement of the right hemisphere in multiplication and addition. In particular, the authors reported an influence over simple multiplication in eight areas of the right hemisphere. Moreover, using the same technique, other authors reported in a single case study that stimulation of the right inferior parietal lobule impaired performance on simple subtraction problems (Yu et al., 2011). The present study thus confirmed and extended previous research by providing neuropsychological data in support of the role of right-hemisphere areas during mental calculation, in particular subtraction and multiplication.

In contrast with our data, however, the study by Yu et al. (2011) also showed that multiplication was not affected by the cortical electrical stimulation on the right parietal or temporal cortex. Yu et al. interpreted their results in light of the hypothesis that an auditory-verbal code (recruiting the left, not the right hemisphere) is used for strategies involving retrieval of information learned by verbal rote memory during school-years (Dehaene, 1992). Nevertheless, alternative strategies may prevail, especially in adult participants whose school-mathematics skills (such as multiplication tables) are generally not exercised nor examined in a common daily basis. In fact, the suggestion has been made that visuo-spatial imagery consists of one of several strategies among which subjects solve arithmetic problems (Siegler and Lemaire, 1997) or maintain numerical information in an active state while solving mental operations (Seron et al., 1992; Noël and Seron, 1993). For instance, previous research indicates that efficient calculation (e.g., the selection of 56 as the correct product to 7 × 8) involves scanning correct products as well as cohort candidate solutions (e.g., the table errors 49 and 54), which may require visuo-spatial abilities (Campbell, 1994; Galfano et al., 2003). This is consistent with the qualitative analysis of multiplication errors of the present study, evidencing that the majority of such errors were inside the same multiplication table. Thus, RHD patients' seemed to have more difficulties on selecting the correct result among the candidate solutions than on accessing the multiplication tables *per se*. In fact, a previous study suggested that TMS applied to the right ventral intra-parietal sulcus impairs this automatized scanning process that supports mental multiplication (Salillas et al., 2012). Moreover, this ability seems to be preserved in left hemisphere (Varley et al., 2005) and impaired in RHD patients (Granà et al., 2006).

Additional indication of the involvement of visuo-spatial processes in mathematical processes comes from a recent study showing a functional and anatomical overlap in the regions implicated in ordering judgments and symbolic calculation (Knops and Wilmes, 2014), and by a study showing interference of both motion perception and numerical comparison when applying TMS on the parietal regions (Salillas et al., 2009). Crucially, the neural circuits described in these two studies were circumscribed to the right hemisphere, which is compatible with the interpretation that visuo-spatial strategies (and not only verbal ones) might be recruited—without success—in our LN patients while solving simple subtraction and multiplication operations.

Our study, however, does not provide data for distinguishing whether the observed mathematical deficits are due to a generic deficit (e.g., in attention or working memory) in RHD patients, or rather to calculation-specific disturbances. Previous literature relates the functioning of the right hemisphere with numerical cognition but also with general visuo-spatial and cognitive processes. For instance, activation of a fronto-parietal network in the right hemisphere is associated with the visuo-spatial component of working memory (Baddeley, 2007), which may play a significant role in arithmetic (Lee and Kang, 2002; DeStefano and LeFevre, 2010). Moreover, right-parietal regions are also activated by shifts of spatial attention (Gitelman et al., 1999; Corbetta et al., 2000), which may be also recruited while solving mental operations (Knops et al., 2009; Dormal et al., 2014).

In the present study we found a correlation between measures of spatial attention and some subtests of the Numerical battery. In particular, we found a robust correlation between spatial attention and odd/even judgment, recognition of arithmetical operations, and oral repetition. No correlation was found instead with simple mental operations or number dictation, which distinguished the patients from controls in our study. These data anticipate a calculation-specific deficit (not mediated by visual-spatial abilities or spatial attention) affecting RHD patients. Still, null effects in correlation analysis should be carefully interpreted in small samples. Thus, further studies looking at the effects of other general-purpose processes, and with a larger sample, should contribute to better characterize RHD patients and their numerical deficits. Moreover, additional studies are currently underway to include more updated and sophisticated neuroimaging analyses to this line of research.

## Conclusion

Our study examined whether basic calculation and number processing would be affected by the presence of LN after right-hemisphere damage. This is the first study to directly address basic calculation abilities (i.e., mostly orally presented tasks) in RHD patients with and without LN, completing the picture provided by previous studies on numerical processing and number representation in these kind of patients. The results showed that LN is associated with a deeper and more specific pattern of mathematical disability, mostly impairing writing abilities as well as mental subtraction and multiplication. RHD patients without LN also showed mathematical disabilities when compared with healthy controls. Yet, the errors in this group were not consistent among individuals.

On the whole, our findings support the hypothesis that numerical skills are impaired in RHD patients, independently of the presence of LN. How does LN precisely determine errors even in simple calculation tasks is hard to understand and could only be matter of speculation before further research is conducted to this aim. The nature of the relation between this impairment and other general-purpose cognitive processes remains awaiting future investigations.

### Conflict of interest statement

The authors declare that the research was conducted in the absence of any commercial or financial relationships that could be construed as a potential conflict of interest.
