# Supplementary material for: Right-hemisphere (spatial?) acalculia and the influence of neglect
Source: Front Hum Neurosci. 2014 Aug 20;8:644. doi: 10.3389/fnhum.2014.00644 (PMC4138500; doi:10.3389/fnhum.2014.00644)
Supplement: Supplementary file 1 [file DataSheet1.PDF]

Appendix 1. Numerical Screening Battery

Task	Items
Verbal counting	From 1-20 and from 20-1 one by one<br>From 2-20 in two by two (e.g. 2,4,6,etc.)<br>From 3-21 in two by two (e.g. 3,5,7,etc.)
Odd/even judgment	77; 81; 21; 9; 52; 20; 4; 43; 18; 86; 467; 3; 70; 74; 156; 6; 35; 12; 11; 15; 69; 28
Number Magnitude Comparison	7-6; 12-18; 13-31; 4-9; 27-70; 49-60; 3-1; 105-500; 220-204; 5-8; 1001-2000; 9100-900; 2-0; 13000-20000
Writing Arabic numerals to dictation	5; 8; 11; 17; 14; 80; 64; 13000; 3400; 12800; 83; 620; 155; 1900; 6010; 10; 8; 3; 2; 5; 7; 4; 6; 9; 1
Reading Arabic numerals	10; 7; 4; 9; 1; 3; 6; 8; 2; 5; 18; 13; 11; 62; 51; 38; 83; 960; 469; 376; 8270; 42300
Recognition of arithmetical operations	3+4; 12-6; 20x4; 32/ 8
Mental one-digit multiplication	8x2; 3x3; 5x6; 0x7; 7x5; 3x9; 1x6; 9x4; 2x2; 7x9
Mental one-digit addition	2+3; 3+4; 4+5; 6+1; 5+2; 3+6; 2+1; 6+5; 7+4; 3+9
Mental one-digit subtraction	2-1; 3-2; 4-2; 5-2; 6-1; 7-4; 8-5; 9-4; 4-0; 8-8
Oral repetition	5; 17; 13; 29; 41; 105; 240; 2106; 9703; 13000; 2; 7; 9; 3; 5; 8

Instructions

Verbal counting	Please, count aloud from...to...
Odd/even judgment	Please, tell me whether the number I am going to say is odd or even
Number Magnitude Comparison	I am going to tell you two numbers. Please tell me which one is larger
Writing Arabic numerals to dictation	Please, write down the Arabic numerals corresponding to the numbers that I will tell you
Reading Arabic numerals	Please, read aloud the number I will show you
Recognition of arithmetical operations	I will tell you some arithmetic operations. Please, tell me which operation is it.
Mental one-digit operations (multiplication, addition, subtraction)	I will tell you some operations. Please, calculate them mentally and tell me the result.
Oral repetition	I will tell you some numbers. Please, repeat them aloud.

Scoring

Each correct answer was scored with one point. The maximum score in each task corresponds to the number of items. Immediate self-corrections were counted as correct answer.
